# Combination therapy with spesolimab and apremilast for refractory generalized pustular psoriasis: a case report

**DOI:** 10.3389/fmed.2025.1668675

**Published:** 2025-08-29

**Authors:** Yuwei Li, Zhenyu Li, Xinhong Hu, Tianyu Cao, Ling Liu

**Affiliations:** ^1^Department of Dermatology, Tangdu Hospital, Fourth Military Medical University, Xi’an, Shaanxi, China; ^2^Department of Urology, Tangdu Hospital, Fourth Military Medical University, Xi’an, Shaanxi, China

**Keywords:** generalized pustular psoriasis, spesolimab, apremilast, IL-36 inhibitors, combination therapy

## Abstract

Generalized pustular psoriasis (GPP) is a rare, life-threatening neutrophilic dermatosis characterized by recurrent episodes of widespread sterile pustules, systemic inflammation, and potential multi-organ failure. We present a 75-year-old woman with a 10-year history of GPP refractory to conventional therapies, including cyclosporine, tripterygium glycosides, and acitretin. Following initial spesolimab infusions that controlled acute flares but failed to prevent relapse, a sequential therapeutic strategy was initiated: a single 900-mg intravenous dose of spesolimab followed by adjunctive apremilast (30 mg twice daily). Within 36 h, her fever subsided (from 39 °C to 36.5 °C), and pustules resolved completely within one week. Notably, apremilast monotherapy maintained sustained remission for 13 months after spesolimab discontinuation, with no adverse events observed. The patient reported significantly restored quality of life and satisfaction with the treatment. This case suggests that combining an anti-interleukin-36 (IL-36) inhibitor for acute control with phosphodiesterase 4 (PDE4) inhibition for maintenance may offer a promising strategy for refractory GPP, though larger studies are needed to validate this approach.

## 1 Introduction

Generalized pustular psoriasis (GPP) is a rare, life-threatening neutrophilic dermatosis characterized by recurrent episodes of widespread sterile pustules, systemic inflammation, and multiorgan failure, with mortality rates of 3%–9% (1, 2). Unlike plaque psoriasis, GPP is driven by dysregulation of the IL-36 signaling pathway, leading to uncontrolled neutrophil recruitment, keratinocyte hyperproliferation, and pro-inflammatory cytokine storms (3). While biologics have demonstrated efficacy in treating GPP, long-term disease management remains challenging, with limited supporting evidence. Case reports suggest that combination therapy—integrating biologics with small-molecule agents—may represent a viable option for GPP patients exhibiting an inadequate response to biologic monotherapy. However, robust clinical data on such therapeutic approaches remain scarce.

To enable dermatologists to develop effective long-term management strategies for these patients, further research and clinical evidence are critically needed.

## 2 Case description

The patient is a 75-year-old female with a 10-year history of GPP. Over her disease course, she sequentially received multiple systemic therapies (Table 1): cyclosporine (3 mg/kg/day for 8 months), a calcineurin inhibitor targeting T-cell activation; compound glycyrrhizin (150 mg/day for 6 months), an anti-inflammatory agent containing glycyrrhizic acid that has immunoregulatory, antiallergic, and hepatoprotective functions, commonly used in Asian GPP patients with hepatic comorbidities (4); tripterygium glycosides (60 mg/day for 3 months), an immunosuppressive botanical extract that suppresses NF-κB and the IL-23/Th17 axis (5); and acitretin (30 mg/day for 4 months), a retinoid that alleviates the condition by regulating cell differentiation and proliferation, exerting anti-inflammatory effects, inhibiting keratin formation, and suppressing neutrophil chemotaxis (6). Despite transient therapeutic responses, all regimens required discontinuation due to disease relapse or adverse events—most notably acitretin-induced cheilitis and palmoplantar desquamation. Compound glycyrrhizin use was based on studies conducted among the Asian population (4), while tripterygium glycosides efficacy data derived from Chinese cohorts (5). Due to poorly controlled skin lesions, significant pain, and recurrent fever, she received a 900 mg intravenous infusion of spesolimab in August 2023 and induced remission for 6 months. Within 48 h post-infusion, her body temperature decreased from 39.2 °C to 36.5 °C, and pustules showed significant resolution within 7 days. In February 2024, the patient experienced a disease relapse and sought treatment at a local traditional Chinese medicine clinic. She received an unspecified traditional Chinese medicine regimen that yielded no significant clinical improvement.

**TABLE 1:** Treatment timeline and clinical outcomes.

Time period	Intervention	Dose/regimen	Clinical outcome	Duration of effect
2014–2023 (approx.)	Cyclosporine	3 mg/kg/day	Transient response; discontinued due to relapse	8 months
	Compound glycyrrhizin	150 mg/day	Partial response; discontinued due to relapse	6 months
	Tripterygium glycosides	60 mg/day	Partial response; discontinued due to relapse	3 months
	Acitretin	30 mg/day	Partial response; discontinued due to cheilitis and palmoplantar desquamation	4 months
August 2023	**Spesolimab (1st infusion)**	900 mg IV single dose	Fever resolved (48 h); pustules resolved (7 days)	6 months remission
February–April 2024	Traditional Chinese medicine	Unspecified	No significant clinical improvement	–
May 2024	**Spesolimab (2nd infusion)**	900 mg IV single dose	Fever resolved (72 h); pustules resolved (5 days)	–
May 2024–Present	**Apremilast**	30 mg twice daily	Sustained remission; no relapse	13+ months

The bold text indicates an ongoing treatment period at the time of manuscript preparation, representing a minimum duration of sustained remission and satisfactory therapeutic effect.

**FIGURE 1:**
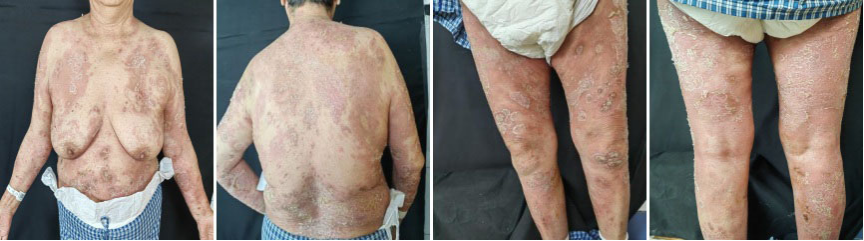
Baseline clinical presentation of generalized pustular psoriasis skin lesions before spesolimab treatment.

**FIGURE 2:**
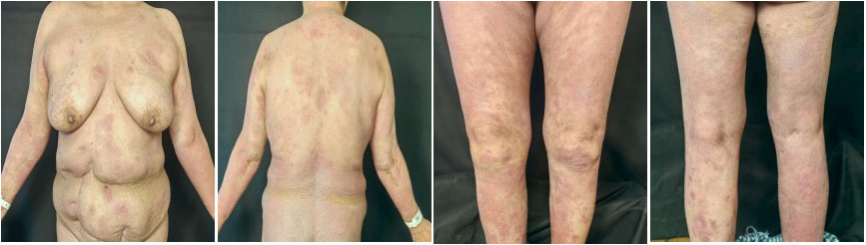
Cutaneous manifestations in the GPP patient 1-week after combination therapy with spesolimab and apremilast.

**FIGURE 3:**
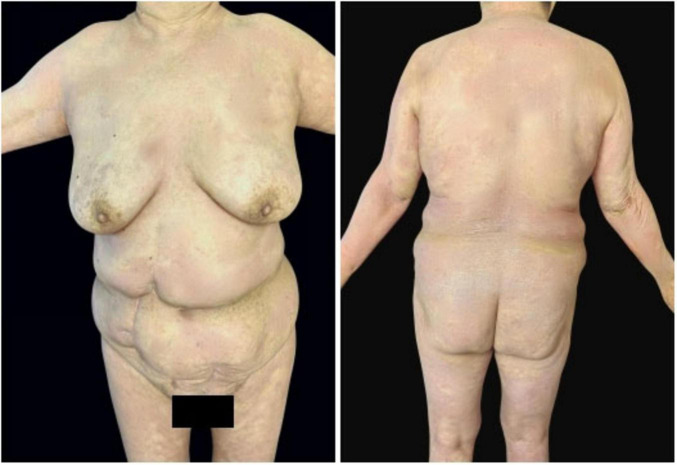
Sustained disease control for 13 months on apremilast monotherapy after spesolimab discontinuation.

In May 2024, following a disease exacerbation (Figure 1), she received another 900 mg intravenous spesolimab infusion. Fever remission occurred within 72 h, and pustules substantially subsided within 5 days (Figure 2). Given the absence of significant comorbidities in this patient, the decision to incorporate apremilast (30 mg twice daily) into her ongoing spesolimab maintenance regimen was informed by therapeutic drug monitoring, laboratory assessments, clinical experience, and literature evidence confirming apremilast-biologic compatibility in psoriasis patients with secondary treatment failure (7–9). Following this combination therapy, her skin lesions demonstrated significant improvement. The patient has now remained spesolimab-free for 13 months while maintained solely on apremilast monotherapy, with sustained disease control throughout this period (Figure 3). No adverse effects, including common gastrointestinal symptoms have been reported, and all laboratory parameters remain within normal limits.

## 3 Discussion

GPP is a rare and severe inflammatory skin disorder characterized by recurrent, widespread, sterile pustules and erythema. It is frequently associated with systemic manifestations, including fever, arthralgia, and leukocytosis. This disorder is underpinned by dysregulation of the IL-36 signaling pathway, which plays a pivotal role in its pathogenesis. This pathway orchestrates a self-amplifying inflammatory cascade: unchecked IL-36 activation stimulates keratinocyte production of CXCL1/IL-8, driving massive neutrophil recruitment that manifests as sterile pustules. GPP can precipitate life-threatening complications, such as multiple organ dysfunction syndrome involving hepatic and renal failure, cardiac failure, and sepsis. These sequelae severely compromise patients’ physical and mental health and quality of life, posing considerable management challenges (10). Racial heterogeneity influences clinical phenotypes: Asian patients exhibit earlier onset, more scalp/nail involvement, and shorter pustular prodromal phases (11).

Conventional GPP management relies on non-targeted immunosuppressants with suboptimal risk-benefit profiles. Retinoids (e.g., acitretin) and cyclosporine achieve 30–40% acute response rates but incur high relapse risks (60% within 48 weeks) and dose-limiting toxicities (12). These agents incur significant toxicities: acitretin causes teratogenicity and mucocutaneous toxicity in 73% of patients, while cyclosporine reduces eGFR by > 30% in 45% of recipients after six months (1, 13).

TNFα, IL-17, and IL-23 inhibitors disrupt the inflammatory cascade driving GPP by targeting key cytokines. TNFα inhibitors (such as infliximab and adalimumab) function by selectively binding and neutralizing soluble and transmembrane TNFα (14). This inhibition attenuates downstream inflammatory cascades, including neutrophil recruitment to the skin, aberrant keratinocyte activation and hyperproliferation, and the production of IL-23 and IL-17, culminating in the rapid resolution of pustules (15). Direct IL-17A inhibitors (e.g., secukinumab, ixekizumab) antagonize the bioactivity of IL-17A, a key effector cytokine (16). By blocking IL-17A signaling through its receptor on keratinocytes, these agents prevent the downstream release of neutrophil-chemoattractant chemokines (notably CXCL1, CXCL8) and antimicrobial peptides (e.g., LL-37), thereby inhibiting neutrophil chemotaxis and subsequent pustule formation (17). IL-23 inhibitors (such as guselkumab and risankizumab) target the p19 subunit, preventing IL-23 engagement with its receptor on T helper cells (18). This suppression of IL-23 signaling critically impairs the differentiation, expansion, and survival of Th17 cells, the primary source of IL-17A and IL-17F, thus interrupting the core pathogenic loop at its origin and promoting sustained disease remission (19). Collectively, these biologic agents disrupt the self-perpetuating inflammatory circuit wherein TNFα promotes dendritic cell-mediated IL-23 release, leading to Th17 cell expansion, excessive IL-17 production, keratinocyte dysregulation, and neutrophil-driven pustule development.

Spesolimab, a humanized anti-interleukin-36 receptor (IL-36R) monoclonal antibody, rapidly resolves acute flares by blocking downstream NF-κB/MAPK activation. The pivotal Effisayil 1 trial demonstrated significant efficacy, with 54% of spesolimab-treated patients achieving pustular clearance within one week compared to 6% on placebo (12). For flare prevention, Effisayil 2 showed 84% relapse-free survival at 48 weeks with quarterly spesolimab (20). Following its initial FDA approval in September 2022 for treating GPP flares in adults, the indication was extended to adolescents (≥ 12 years, ≥ 40 kg) in 2024 (21). Comparatively, imsidolimab, an anti-IL-36γ monoclonal antibody, exhibits superior efficacy in IL36RN-wild-type patients, achieving 79% pustule clearance at week 4 (22), attributed to its selective neutralization of the most abundant IL-36 isoform within GPP pustules. Despite these advancements and the introduction of targeted biologics like spesolimab, a substantial proportion of GPP patients still experience inadequate responses or relapses, leading to refractory disease. Consequently, maintaining long-term remission remains a critical challenge, as residual inflammatory activity can trigger disease relapse.

Apremilast, an oral PDE4 inhibitor, modulates excessive keratinocyte proliferation and neutrophil infiltration by targeting intracellular signaling pathways involved in pro-inflammatory cytokine and chemokine production (23, 24). Beyond its established anti-Th17 effects, apremilast directly targets neutrophilic inflammation through PDE4 inhibition. Elevated cyclic adenosine monophosphate (cAMP) activates protein kinase A (PKA), which suppresses extracellular signal-regulated kinase/c-Jun N-terminal kinase (ERK/JNK) phosphorylation and calcium flux, thereby inhibiting reactive oxygen species (ROS) generation, neutrophil extracellular trap formation (NETosis), and CD11b/CD18-mediated adhesion. Transcriptomic analyses further demonstrate downregulation of chemotaxis and innate immunity genes (e.g., *IL-8*, *CXCR1*, *CCL3*), reducing neutrophil recruitment to inflammatory sites. Approved for treating various inflammatory skin conditions, including psoriasis vulgaris, apremilast is indicated for the maintenance therapy of GPP. Following induction therapy with spesolimab, apremilast can help maintain remission not only through inhibition of residual Th17 pathway activity but also by ameliorating neutrophil-driven tissue damage via the above mechanisms. This pathway critically contributes to psoriasis pathogenesis, and its persistent activation is implicated in the refractory nature of some GPP cases (25).

Our therapeutic strategy leverages phase-specific pathophysiology in GPP. During acute flares, spesolimab—a humanized IL-36R monoclonal antibody—rapidly interrupts the IL-36-driven hyperinflammation within 48 h by blocking IL-36R signaling, effectively clearing pustules and reducing disease severity through inhibition of the overactive IL-36 pathway, a key driver of inflammation and keratinocyte hyperproliferation in GPP (3, 26). The rationale for transitioning to apremilast monotherapy for maintenance aligns with emerging evidence on sustained GPP control. While the Effisayil 2 trial demonstrated spesolimab’s efficacy in flare prevention (26), continuous IL-36 inhibition may not be necessary for all patients. In our case, apremilast maintenance leveraged its dual mechanism: suppressing residual Th17 activity while targeting neutrophil chemotaxis—a critical pathway in GPP relapse. This sequential approach optimizes long-term safety given apremilast’s favorable tolerability profile. Spesolimab has demonstrated rapid and significant efficacy in clearing pustules and reducing disease severity during GPP flares by effectively inhibiting the overactive IL-36 signaling pathway, a key driver of inflammation and keratinocyte hyperproliferation in GPP.

This case demonstrated a robust initial response to spesolimab induction therapy, achieving marked reduction in pustular eruptions and systemic symptoms. However, relapse occurred several months later. Given inadequate response to conventional therapies (cyclosporine, tripterygium glycosides, compound glycyrrhizin, acitretin) and significant adverse effects during acitretin treatment, we implemented a sequential biologic strategy: spesolimab for acute flare control with transition to apremilast for maintenance therapy. This approach exploits complementary mechanisms—spesolimab targets the acute IL-36-driven inflammatory cascade, while apremilast modulates the chronic Th17-mediated inflammatory milieu. Subsequent apremilast initiation proved essential for sustaining remission and preventing relapse. The combination demonstrated a favorable safety profile, with no treatment-emergent adverse events observed during the monitoring period. This tolerability is clinically significant given the requirement for long-term therapy in GPP.

In conclusion, our case supports the therapeutic potential of sequential spesolimab-apremilast therapy for refractory GPP. By concurrently targeting distinct pathogenic pathways (IL-36 hyperinflammation and Th17 dysregulation), this strategy may offer enhanced efficacy and durability. Further validation through larger cohort studies and randomized controlled trials is warranted to define optimal dosing and treatment duration. Such investigations could establish new paradigms for managing this debilitating disease. Ultimately, such research may pave the way for new treatment paradigms that can improve outcomes for patients with this challenging and debilitating disease.

## Data Availability

The original contributions presented in this study are included in this article/supplementary material, further inquiries can be directed to the corresponding author.
